# Cardioprotective Effects of Voluntary Exercise in a Rat Model: Role of Matrix Metalloproteinase-2

**DOI:** 10.1155/2015/876805

**Published:** 2015-03-22

**Authors:** Anikó Pósa, Renáta Szabó, Krisztina Kupai, Zoltán Baráth, Zita Szalai, Anett Csonka, Médea Veszelka, Mariann Gyöngyösi, Zsolt Radák, Rudolf Ménesi, Imre Pávó, Anikó Magyariné Berkó, Csaba Varga

**Affiliations:** ^1^Department of Physiology, Anatomy and Neuroscience, University of Szeged, Kozep Fasor 52, Szeged 6726, Hungary; ^2^Faculty of Dentistry and Department of Orthodontics and Pediatric Dentistry, University of Szeged, Szeged 6720, Hungary; ^3^Department of Cardiology, Medical University of Vienna, Waehringer Guertel 18-20, 1090 Vienna, Austria; ^4^Institute of Sport Science, Faculty of Physical Education and Sport Science, Semmelweis University, Alkotas Ucta 44, Budapest 1123, Hungary

## Abstract

*Background*. Regular exercise at moderate intensity reduces cardiovascular risks. Matrix metalloproteinases (MMPs) play a major role in cardiac remodeling, facilitating physiological adaptation to exercise. The aim of this study was to examine the influence of voluntary physical exercise on the MMP-2 enzyme activity and to investigate the cardiac performance by measurement of angina susceptibility of the heart, the basal blood pressure, the surviving aorta ring contraction, and the cardiac infarct size after I/R-induced injury. *Methods*. Male Wistar rats were divided into control and exercising groups. After a 6-week period, the serum level of MMP-2, basal blood pressure, cardiac angina susceptibility (the ST segment depression provoked by epinephrine and 30 s later phentolamine), AVP-induced heart perfusion and aorta ring contraction, infarct size following 30 min ischemia and 120 min reperfusion, and coronary effluent MMP-2 activity were measured. *Results*. Voluntary wheel-running exercise decreased both the sera (64 kDa and 72 kDa) and the coronary effluent (64 kDa) MMP-2 level, reduced the development of ST depression, improved the isolated heart perfusion, and decreased the ratio of infarct size. *Conclusion*. 6 weeks of voluntary exercise training preserved the heart against cardiac injury. This protective mechanism might be associated with the decreased activity of MMP-2.

## 1. Introduction

Regular physical exercise has been shown to reduce many cardiovascular risk factors and is associated with a number of cardiovascular benefits. The reduced levels of cardiovascular mortality and cardiac ischemic events in patients who participate in regular exercise training are mainly due to the control of cardiovascular risk factors (e.g., high blood pressure and obesity) [1, 2].

Although regular exercise training has been confirmed as a pragmatic and sustainable countermeasure for cardioprotection, the precise underlying mechanisms for these beneficial effects remain to be defined.

One group of enzymes that is important in mediating the destructive effects of cardiovascular disease is the family of matrix metalloproteinases (MMPs). The MMPs are a large family of calcium-dependent, zinc-containing endopeptidases that have the ability to remodel the extracellular matrix in both physiological and pathological processes. Of this diverse family of enzymes, MMP-2 (also known as gelatinase A) is found in nearly all cell types and plays a key role in the cardiovascular system, ranging from heart development to ischemia/reperfusion (I/R) injury [3]. The release of MMP-2 increases with increasing duration of ischemia and correlates negatively with functional recovery. Over the past few decades, many approaches to the relationship between MMP-2 activity and infarct size have been studied [4]. These investigations have revealed that the inhibition of MMP-2 protects the heart from cardiac dysfunction [5]. However, the activation of MMP-2 during an ischemic insult is associated with a larger infarct size of the heart [6]. Myocardial ischemia followed by reperfusion results in I/R injury characterized by a decreased myocardial function and tissue necrosis which is a marker of a long-term left ventricular function in patients who have suffered an acute myocardial infarction [7]. The purpose of this study was to assess the effects of 6 weeks of voluntary physical exercise on MMP-2 activity and release into the coronary effluent and to investigate the cardiac performance. We additionally measured the angina susceptibility of the heart, the basal blood pressure, and surviving aorta ring contraction and determined the cardiac infarct size after I/R-induced injury.

## 2. Materials and Methods

### 2.1. Animals and Experimental Design

All experimental procedures were performed in accordance with the standards of the European Community guidelines on the care and use of laboratory animals and had been approved by the local Institutional Ethics Committee at the University of Szeged.

Male Wistar rats (*n* = 70, weighing 200–230 g; Toxi-Coop Zrt., Hungary) were randomly assigned to control and exercising groups. The exercising animals were placed individually into cages fitted with a running wheel (Acellabor Ltd., Budapest, Hungary) and were allowed free access to the wheel for 24 h per day for 6 weeks [8]. The exercising protocol, defined as a voluntary wheel-running model, was selected in an effort to isolate the effects of exercising from the additional stress associated with forced exercise protocols. During the exercising period, the average running distance was 3.91 ± 1.27 km/day/animal. Control rats were placed in standard holding cages without a running wheel for the same period. All animals were housed in a temperature-controlled facility (23°C) maintained on a 12:12 h light-dark cycle with food and water provided* ad libitum*.

### 2.2. Measurement of MMP-2 Activity

Metalloproteinase activity was detected by gelatin zymography. Sodium dodecyl sulfate-polyacrylamide gels (8%) were copolymerized with 20 mg/mL gelatin. A constant amount of protein was loaded in each well under nonreducing conditions and was separated by electrophoresis. After electrophoresis, the gels were washed with 2.5% Triton X-100 and incubated overnight in incubation buffer (50 mM Tris-HCl, 150 mM NaCl, 5 mM CaCl_2_, and 0.05% NaN_3_) at 37°C. After staining with 0.05% Coomassie Brilliant Blue in a mixture of methanol/acetic acid/water and being destained with aqueous 4% methanol/8% acetic acid, enzyme activity was detected as colorless bands against the blue-stained background. A protein ladder (Spectra Multicolor Broad Range Protein Ladder, Thermo Scientific) was used to identify the individual enzymes (MMP-2, 72 kDa and 64 kDa isoforms) for molecular weights. Zymograms were scanned digitally and the band intensities were quantified by using Quantity One software (Bio-Rad, Hercules, CA, USA).

### 2.3. Measurement of Basal Blood Pressure

The animals were anaesthetized with 30.0% urethane (0.50 mL/100 g, i.p.) and the mean arterial blood pressure was measured in the right carotid artery, expressed as mmHg. The right carotid artery was separated from the vagus nerve and clamped with a bulldog clamp for cannulation. Then the carotid artery cannulation was connected to the HAEMOSYS computerized complex hemodynamic analysis system (Experimetria UK, London) to record the mean blood pressure. After 10–15-minute stabilization period, the baseline recording has carried out over a 10-minute period to examine the mean blood pressure. The core temperature of the rats was maintained at 37°C with a homeothermic control unit (Harvard Instrument, UK).

### 2.4. Experimental Angina Provoked by Epinephrine Plus Phentolamine

The animals were anesthetized with 30.0% urethane (0.50 mL/100 g, i.p.) and the standard limb lead II of the surface electrocardiogram (ECG) was recorded by the HAEMOSYS system and expressed as mV. The change in ST segment was measured and used as the index of angina severity. The mean ECG voltage 13 ms after the peak of the S wave was defined as the value of the ST segment, as described previously [9]. In the epinephrine plus phentolamine model, single doses of epinephrine (10.0 *μ*g/kg) and 30 s later the *α*-adrenoreceptor antagonist phentolamine (15.0 mg/kg) were administered into the tail vein of the rat. Each agent was dissolved in 0.20 mL of physiological saline and injected over 2 s. To measure the difference in the amplitude of ST segment, the ECG recording has been carried out from the challenge of epinephrine over a 10-minute period.

### 2.5. Measurement of Arginine Vasopressin- (AVP-) Induced Heart Perfusion according to Langendorff

After cervical dislocation, the hearts were rapidly excised and then perfused according to Langendorff at a constant pressure of 75 mmHg with Krebs-Henseleit buffer (118.0 mM NaCl, 4.70 mM KCl, 2.50 mM CaCl_2_, 1.18 mM MgSO_4_, 25.0 mM NaHCO_3_, 1.18 mM KH_2_PO_4_, and 5.50 mM glucose), gassed with a mixture of 95% O_2_/5% CO_2_ at 37°C. Following the cannulation, the heart perfusion in response to AVP was measured. The AVP quantity (0.01, 0.1, and 1.0 *μ*g) was added in Krebs-Henseleit buffer and then 0.5 mL of AVP-Krebs solution was injected into the Langendorff apparatus by way of a valve attached to the cannula. We administrated the perfusion changes every 30 s for 5 min. Each dose of AVP was injected after a 15-minute stabilization period. Data are expressed as a percentage of the decrease relative to the basal value.

### 2.6. Measurement of Surviving Aorta Contraction

The rats were killed by cervical dislocation, and the abdominal aortas were removed and placed in chilled Krebs-Henseleit solution (118.0 mM NaCl, 4.70 mM KCl, 2.50 mM CaCl_2_, 1.18 mM MgSO_4_, 25.0 mM NaHCO_3_, 1.18 mM KH_2_PO_4_, and 5.50 mM glucose) which was gassed with 95% O_2_ and 5% CO_2_. The aorta contraction was measured as described previously [10]. Briefly, the aortas were cleaned of all adipose and connective tissue, the abdominal region was cut into rings (3 mm long), and their weights were measured. To induce the contraction changes, we freshly added a 2 *μ*g/mL dose of AVP into the incubation buffer [10]. The isometric tension was measured through the transducer, which was connected to an ISOSYS computerized program system (Experimetria, UK, London) for continuous recording of the blood vessel tension. The contractile response to vasopressin was expressed in terms of the tension of the aorta ring (g/mg ring weight).

### 2.7. Ischemia/Reperfusion Protocol

After cervical dislocation the hearts were rapidly excised and then perfused in Langendorff mode at a constant pressure of 75 mmHg with Krebs-Henseleit buffer, gassed with a mixture of 5% CO_2_  + 95% O_2_ at 37°C. After normoxic perfusion for 10 min, local ischemia was induced by occlusion of the left anterior descending coronary artery (LAD) for 30 min. This was followed by reperfusion for 120 min. The coronary effluent was collected during the first 5 min of reperfusion and was concentrated in Amicon Ultra Centrifugal concentrating vessels (5000 g, 4°C, Millipore, MA, USA) for MMP-2 activity determination via gelatin zymography. At the end of the protocol the hearts were stained with 1% Evans blue and then were frozen at −20°C overnight.

### 2.8. Infarct Size Determination

Infarct size was measured after regional ischemia induced by LAD occlusion* ex vivo* [11]. Frozen hearts were cut into 2 mm thick cross-sectional slices. These slices were stained with 1% 2,3,5-triphenyltetrazolium chloride (TTC) for 10 min at 37°C. After TTC staining, the slices were transferred to a formalin (10%) solution for 10 min and then placed in phosphate buffer (pH = 6). Following this incubation, both sides of each slice were photographed with a digital camera. Infarct size was calculated as the percentage of the area at risk.

### 2.9. Statistics

The results shown in the figures are expressed as means ± S.E.M. Differences between groups were determined with two-tailed Student's *t*-test and *P* values less than 0.05 were considered significant.

## 3. Results

### 3.1. Sera 64 kDa and 72 kDa MMP-2 Activity

**Figure 1:**
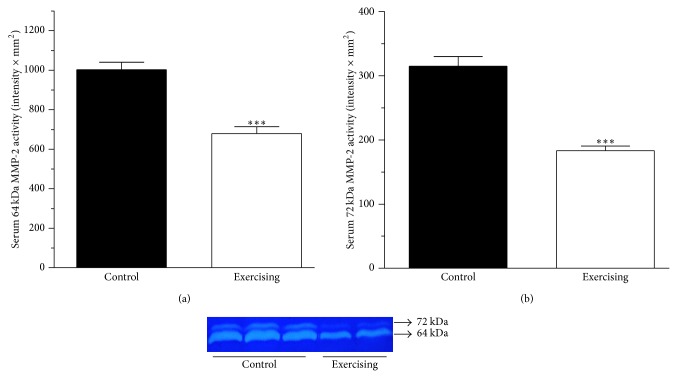
Serum matrix metalloproteinase-2 activity (64 kDa and 72 kDa MMP-2, expressed as intensity × mm^2^) in control (■) and exercising (□) rats. Data are means ± SEM, *n* = 12-13. Statistical significance: ^***^ *P* < 0.001 as compared with the control group. (a) Serum 64 kDa matrix metalloproteinase-2 activity (64 kDa MMP-2, expressed as intensity × mm^2^) in control (■) and exercising (□) rats. (b) Serum 72 kDa matrix metalloproteinase-2 activity (72 kDa MMP-2, expressed as intensity × mm^2^) in control (■) and exercising (□) rats. The representative image presents a zymographic picture.

Serum samples were collected from the lateral tail vein. We found that the 6-week voluntary physical exercise significantly (*P* < 0.001) decreased the serum MMP-2 activity (that of 64 kDa MMP-2 from 1002.71 ± 37.50 to 679.73 ± 34.35 intensity × mm^2^ and that of 72 kDa MMP-2 from 314.93 ± 14.80 to 183.33 ± 7.12 intensity × mm^2^, *n* = 12-13). Data are shown in Figure 1.

### 3.2. Measurement of Basal Blood Pressure

**Figure 2:**
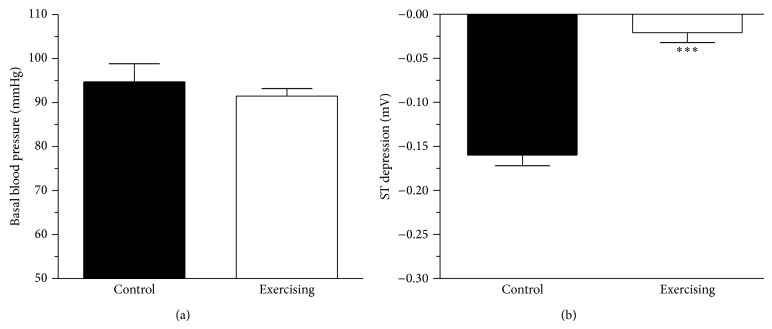
(a) The basal blood pressure (mmHg) in control (■) and exercising (□) animals. Data are expressed as means ± S.E.M., *n* = 14–18. (b) The effect of recreational physical exercise on ST segment changes (measured in a lead II standard surface ECG; expressed in mV) following intravenous injection of epinephrine (10.0 *μ*g/kg) and 30 s later phentolamine (15.0 mg/kg). Data are shown as means ± S.E.M, *n* = 14–18. Statistical significance: ^***^ *P* < 0.001 as compared with the control group.

The basal blood pressure measured in the right carotid artery proved to be similar in the control and exercising rats (from 94.71 ± 4.13 to 91.48 ± 1.69, *n* = 14–18). Data are presented in Figure 2(a).

### 3.3. Experimental Angina Provoked by Epinephrine Plus Phentolamine

The administration of phentolamine 30 s after epinephrine caused a robust (*P* < 0.001) ST segment depression in the control rats (−0.16 ± 0.012 mV, *n* = 14–18). As a result of 6-week physical exercise we have found an improvement in ST segment changes (−0.021 ± 0.011 mV, *n* = 14–18, *P* < 0.001). Data are shown in Figure 2(b).

### 3.4. Measurement of Isolated Heart Perfusion Provoked by AVP

**Figure 3:**
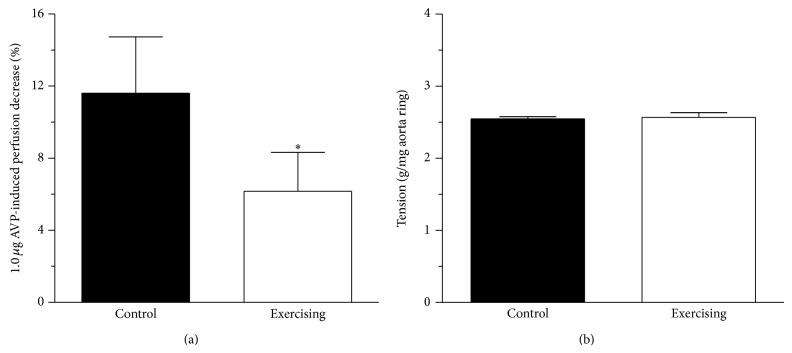
(a) The Langendorff heart perfusion decrease expressed as a percentage in response to 1.0 *μ*g arginine-vasopressin (AVP) in control (■) and exercising (□) rats. Means ± S.E.M., *n* = 8–10. Statistical significance: ^*^ *P* < 0.05 as compared with the control group. (b) The effect of 2.0 *μ*g AVP-induced aorta ring contraction (expressed as g/mg aorta ring weight) on control (■) and exercising (□) animals. Results are shown as means ± S.E.M., *n* = 9-10.

The perfusion measured according to Langendorff is illustrated in Figure 3(a). No differences in basal perfusion or in response to 0.01 or 0.1 *μ*g AVP were observed between the control and the exercising animals. However, 1.0 *μ*g AVP revealed a significantly (*P* < 0.05) improved perfusion in the exercising group (from 11.6 ± 3.14 to 6.17 ± 2.16%, *n* = 8–10).

### 3.5. Measurement of Aorta Contraction Provoked by AVP

Results observed in the experiment involving the surviving aorta ring contraction are demonstrated in Figure 3(b). In response to the 2.0 *μ*g/mL dose of AVP, we found no significant difference between the control and the exercising animals (from 2.55 ± 0.03 to 2.57 ± 0.07 g/mg aorta ring, *n* = 9-10).

### 3.6. Determination of Infarct Size

**Figure 4:**
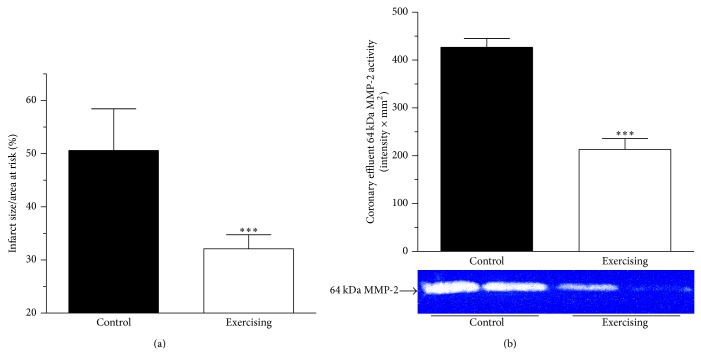
(a) Infarct size is demonstrated as a percentage of the area at risk in control (■) and exercising (□) groups. Means ± S.E.M., *n* = 11-12. Statistical significance: ^***^ *P* < 0.001 as compared with the control animals. (b) Densitometrically assessed MMP-2 activity in coronary effluent collected from isolated perfused hearts during the first 5 min of reperfusion (64 kDa MMP-2, expressed as intensity × mm^2^) in control (■) and exercising (□) rats. Means ± S.E.M., *n* = 10. Statistical significance: ^***^ *P* < 0.001 as compared with the control group.

Figure 4(a) shows the infarct size after 30 min of LAD occlusion and 120 min of reperfusion expressed as a percentage of the area at risk. 6 weeks of voluntary exercise training significantly (*P* < 0.001) reduced the infarct size in the exercising animals as compared with the control group (from 50.6 ± 7.86% to 32.12 ± 2.66%, *n* = 11-12).

### 3.7. Coronary Effluent MMP-2 Activity

The coronary effluent MMP-2 activity was determined after the 30-minute LAD occlusion, during the first 5 min of reperfusion. In the exercising group the activity of the 64 kDa MMP-2 isoform was significantly (*P* < 0.001) decreased from 426.94 ± 18.37 to 213.32 ± 22.45 intensity × mm^2^, *n* = 10). Data are presented in Figure 4(b).

## 4. Discussion

We demonstrated that 6 weeks of voluntary exercise was able to decrease the levels of serum and coronary effluent MMP-2 activity, reduce the myocardial infarct size, and improve the angina susceptibility of the heart. Voluntary wheel-running additionally resulted in an improvement in myocardial perfusion.

We used a voluntary wheel-running model where the animals were able to self-select the time, duration, and intensity of exercise in a nonstressful environment. Previously, 3-4 weeks of voluntary wheel-running had been demonstrated to induce robust physiological hypertrophy, which provokes a beneficial adaptive response of the cardiovascular system [12, 13]. We therefore employed a 6-week exercise protocol to provide sufficient stimuli for adaptation.

Regular physical exercise results in physiological left ventricular hypertrophy which contributes to several cardiovascular benefits. Resting bradycardia has been considered to be the hallmark cardiovascular effect of exercise-training adaptation [14, 15]. Changes after regular exercise might result in an improvement in bioenergetics and metabolic status and modification in endogenous defense system. There is a broad consensus that antioxidative mechanisms play a key role in cardioprotection through training-induced upregulation [16, 17]. However, besides the antioxidant defenses, another mechanism, MMP-2 secretion, can be modulated by exercise. Most previous studies have reported data concerning MMP activity after various training models in either the skeletal muscle [18] or the cardiac tissues [19]. Less information is available concerning the influence of exercise-induced MMP-2 in the circulation. To investigate this question, serum and released MMP-2 activity into the coronary effluent were analyzed. Urso et al. measured the serum MMP-2 activity after a single bout of exercise but did not find a change in their study [20]. In our present investigation involving wheel-running animals, we hypothesized that exercise would influence the serum MMP-2 activation. Our zymography analyses revealed that 6 weeks of voluntary exercise training decreased the serum levels of the 64- and 72 kDa MMP-2 forms in the exercising animals as compared with the control group. Previous human studies have demonstrated that physical exercise can affect the levels of MMPs following acute and chronic interventions. Under coronary risk events, the elevations of MMP-2 and MMP-9 are correlated with increased inflammation [21]. The decrease in MMPs could be related to expression of the tissue inhibitors of metalloproteinases (TIMPs), protease degradation, and reduction of proinflammatory cytokine tumor necrosis factor-alpha (TNF-*α*) [22]. The lowering levels of inflammatory biomarkers mediate the inhibitory effect of exercise on MMP-2 and MMP-9 levels [23]. Lucotti et al. have investigated the effects of aerobic exercise and found that the exercise program caused about 20% reduction in TNF-*α* and MMP-2 levels [24]. Thus, the alterations on MMP's circulating concentrations might be a good reflection of the exercise effect on inflammation markers. The decrease in the level of serum MMP-2 is a systemic effect which might be contributing to the multiple adaptation mechanisms of physical exercise.

It is well known that the increased activity of MMP-2 during cardiac I/R contributes to the disruption of the endothelial layer and increase of its secretion into the coronary circulation. Cheung et al. first reported that MMP-2 can be released into the coronary effluent of perfused rat hearts. The release of MMP-2 peaked during the first and fifth minutes of reperfusion and was enhanced with increasing duration of ischemia [25]. In a previous study, Lalu et al. examined the effect of I/R on the gelatinolytic activity of MMP-2 in the heart ventricles and coronary effluent. Gelatinolytic activities of MMP-2 were detected before and after I/R and it was found that the release of MMP-2 into the coronary effluent was higher after I/R. It is known that in the first minutes of reperfusion there is a burst of reactive oxygen species (ROS) which determine the severity of the reperfusion injury [26]. The release of MMP-2 correlates negatively with the functional recovery [27]. Our data demonstrated that 6-week voluntary exercise was protective against reperfusion damage, since we found that the release of 64 kDa MMP-2 into the perfusate decreased significantly in the exercising rats. However, we did not identify the 72 kDa isoform activity in the coronary effluent.

Increased activity of the MMP-2 into the perfusate contributes to the disruption of the endothelial layer and has a negative impact on the vascular permeability and leads to coronary artery disease and heart failure [28]. However, the decrease in infarct size leads to an improved cardiac function [29–33]. Human epidemiological data clearly suggest that regular exercise reduces the risk of death during clinical I/R injury [34]. The results of our study show that 6-week voluntary wheel-running provides protection against I/R injury by reducing the myocardial infarct size. The extent of the necrotic area in exercised hearts was lessened by more than 50%. Doustar et al. found that 4-week resistance training did not protect the heart against I/R-induced injury [35]. The difference in these results could have resulted from methodological differences, such as the type and duration of exercise. The relationship between MMP-2 and infarct size was supported in a large body of different experimental evidences [4, 36, 37]. Our current data support this possible link between released MMP-2 activity and reduced infarct size.

That exercise training resulting in cardiovascular benefits that causes functional and structural adaptations is widely investigated. During exercise, increased blood flow and shear stress augment endothelium dependent vasodilatation through the upregulation of endothelial nitric oxide synthase (eNOS) [28]. The nitric oxide (NO), particularly derived from eNOS, has been implicated in the cardioprotection offered by exercise [38]. Increased arterial dilation induced by NO improves myocardial oxygen supply [39] and may indicate additional endothelium-dependent functions that prevent ischemic events. Exercise-induced NO may be a potential inducer of heme oxygenase (HO) enzyme system. In earlier investigations, many researches demonstrate the beneficial roles of the HO enzyme system in cardiovascular function [10, 40, 41]. In a previous study, Sun et al. found that exercise-induced elevation of vascular HO and enhanced HO-related dilation demonstrate the direct participation of the HO system in cardiovascular adaptation [42]. In a diet-induced obese mouse model, Hafstad et al. showed that the impairment of left ventricular (LV) function and mechanoenergetics were normalized by moderate-intensity training. They demonstrated that the changes were associated with altered myocardial substrate utilization and improved mitochondrial capacity as well as reduced oxidative stress, fibrosis, and intracellular matrix metalloproteinase-2 content [43].

To prove the adaptive and protective effects of voluntary wheel-running exercise, cardiac parameters were measured in this study. We detected the ST segment depression, which is considered a reliable ECG finding and has been associated with a worse prognosis for patients with coronary artery disease [44]. 6 weeks of voluntary exercise diminished the ST segment depression and therefore improved the ischemia susceptibility of the heart.

We have observed that basal normoxic heart perfusion was similar in the exercising group and the control group. We therefore utilized AVP, which can regulate the hemodynamic parameters by inducing moderate vasoconstriction. In response to 1.0 *μ*g AVP, a significant improvement in heart perfusion was observed after exercise training. The AVP-induced perfusion changes imply that a 6-week period of voluntary exercise may be effective in producing functional and structural adaptations in the cardiovascular system.

We also measured the AVP-induced aorta ring contraction and the basal blood pressure. In our earlier study, the surviving aorta ring contraction was measured by incubation with AVP (2.0 *μ*g/mL) in male and female rats [10]. The 2.0 *μ*g/mL AVP dose was earlier the most effective one for the detection of gender differences, so we used this treatment in the present investigation [10]. Despite our results revealing favorable adaptation in the cardiovascular system after voluntary exercise training, no differences in aorta contraction were observed between the control and the exercising animals.

Similarly, no alteration in basal blood pressure was detected between the control and the exercising rats. In another investigation, Roque et al. measured the hemodynamic parameters after swimming training in normotensive rats and also found no blood pressure changes after exercise [45]. The effect of aerobic exercise training on the blood pressure of normotensive animals and humans seems to be minimal and our results are in agreement with those of other studies. Other investigations clearly demonstrated that hypertensive rats and rats with metabolic syndrome had higher blood pressure levels than did normotensive rats in which aerobic exercise did not induce any change in the blood pressure [46, 47].

In this current study, wheel-running exercising decreased the activities of both 64 kDa and 72 kDa MMP-2 in the serum and also the release of MMP-2 from the heart into the coronary effluent as a consequence of 30-minute LAD occlusion. Similar to the decreases in the MMP-2 values, the infarct size was also reduced. Furthermore, such a training period seems to be a potent stimulus for functional recovery in respect of the myocardial perfusion and ischemic susceptibility of the heart.

In conclusion, our results show adaptive and cardioprotective effects of voluntary wheel-running exercise. The reduced activity of serum MMP-2 might be contributing to the multiple adaptation mechanisms of 6-week physical exercise. The fact that the infarct size is decreased suggests that liberation of MMP-2 into the perfusate could be part of cardioprotective effects. Moreover, our training program was able to improve the angina susceptibility of the heart and causes functional recovery detected by AVP-induced perfusion changes.
